# The Parkinson's disease–associated kinase LRRK2 regulates genes required for cell adhesion, polarization, and chemotaxis in activated murine macrophages

**DOI:** 10.1074/jbc.RA119.011842

**Published:** 2020-02-28

**Authors:** Daniel R. Levy, Atul Udgata, Panagiotis Tourlomousis, Martyn F. Symmons, Lee J. Hopkins, Clare E. Bryant, Nicholas J. Gay

**Affiliations:** ‡ Department of Biochemistry, University of Cambridge, Tennis Court Road, Cambridge CB2 1GA, United Kingdom; § Department of Veterinary Medicine, University of Cambridge, Madingley Road, Cambridge CB3 0ES, United Kingdom

**Keywords:** transcriptomics, Parkinson's disease, macrophage, protein kinase, guanine nucleotide exchange factor (GEF)

## Abstract

Leucine-rich repeat kinase 2 (*LRRK2*) encodes a complex protein that includes kinase and GTPase domains. Genome-wide association studies have identified dominant *LRRK2* alleles that predispose their carriers to late-onset idiotypic Parkinson's disease (PD) and also to autoimmune disorders such as Crohn's disease. Considerable evidence indicates that PD initiation and progression involve activation of innate immune functions in microglia, which are brain-resident macrophages. Here we asked whether LRRK2 modifies inflammatory signaling and how this modification might contribute to PD and Crohn's disease. We used RNA-Seq–based high-resolution transcriptomics to compare gene expression in activated primary macrophages derived from WT and *Lrrk2* knockout mice. Remarkably, expression of a single gene, Rap guanine nucleotide exchange factor 3 (*Rapgef3*), was strongly up-regulated in the absence of LRRK2 and down-regulated in its presence. We observed similar regulation of *Rapgef3* expression in cells treated with a highly specific inhibitor of LRRK2 protein kinase activity. *Rapgef3* encodes an exchange protein, activated by cAMP 1 (EPAC-1), a guanine nucleotide exchange factor that activates the small GTPase Rap-1. Rap-1 mediates cell adhesion, polarization, and directional motility, and our results indicate that LRRK2 modulates chemotaxis of microglia and macrophages. Dominant PD-associated *LRRK2* alleles may suppress EPAC-1 activity, further restricting motility and preventing efficient migration of microglia to sites of neuronal damage. Functional analysis *in vivo* in a subclinical infection model also indicated that Lrrk2 subtly modifies the inflammatory response. These results indicate that LRRK2 modulates the expression of genes involved in murine immune cell chemotaxis.

LRRK2 is a large protein of 286 kDa consisting of a complex and unique arrangement of protein–protein interaction and functional domains. This arrangement consists of N-terminal repeats, including ankyrin repeats, a leucine-rich repeat (LRR)^3^ domain, a Ras of complex proteins (Roc) GTPase with an associated C-terminal of Roc domain, a Ser/Thr protein kinase, and a WD40 domain at the C terminus of the protein (1). The presence of a Roc-C–terminal of Roc tandem domain defines LRRK2 as a member of the Roco protein family, a family first detected in the slime mold *Dictyostelium discoideum* (2, 3).

There is great interest in all aspects of LRRK2 biology because genome-wide association studies have identified many variants in this protein that predispose to late-onset Parkinson's disease. The most common mutation in the LRRK2 gene results in a change from glycine to serine at amino acid 2019 (G2019S). This SNP is the highest known risk factor for development of Parkinson's disease, accounting for 5%–7% of autosomal dominant familial cases (4, 5) and 1%–2% of sporadic cases in Western populations (6). Genome-wide association studies have also revealed a link with Crohn's disease and leprosy. Genetic links with these diseases demonstrate a nonneuronal but clearly innate immune component to LRRK2 biology (7).

LRRK2 expression is enriched in macrophages, B cells, and dendritic cells (8). Innate immune stimuli, such as IFN-γ, stimulate LRRK2 expression, revealing responsiveness to activation of innate immune signaling pathways mediated by pattern recognition receptors (9). Furthermore, activation of Toll-like receptors (TLRs) by pathogen-associated molecules such as bacterial lipopolysaccharide (LPS) leads to phosphorylation of LRRK2 by IκB kinase at two serine residues (Ser-910 and Ser-935) (10). The IκB kinase family is normally associated with phosphorylation of IκB proteins that sequester NF-κB in the cytoplasm. Phosphorylation and ubiquitination of IκB proteins leads to proteolysis and subsequent transfer of NF-κB into the nucleus (11). LRRK2 phosphorylation depends on the TLR adaptor (Myd88), an innate immune signal transducer that mediates signaling from cell-surface TLRs, as well as TLR7, TLR8, and TLR9, which signal from the endosomal compartment.

The role of inflammatory processes in the etiology of Parkinson's disease is further illustrated by administration of LPS systemically and directly into the substantia nigra. In the latter case, LPS causes irreversible degeneration of dopaminergic neurons of the pars compacta, observed a week after injection (12, 13). Notably nondopaminergic neurons of the nigrostriatal system, as well as proximal dopaminergic neurons not associated with the nigrostriatal pathway, remain unaffected by direct LPS injection. Therefore, LPS injection and the resulting inflammatory insult demonstrate remarkable sensitivity and specificity to the dopaminergic circuitry associated with Parkinson's disease. In another study, the same pattern of Parkinson's disease–like microglial activation, followed by neurodegeneration over 10 months, was observed when LPS or tumor necrosis factor α were administered systemically in mice via intraperitoneal injection (14). In humans, a laboratory worker accidentally exposed to *Salmonella*-derived LPS developed many symptoms of Parkinson's disease, including bradykinesia, rigidity, and tremor at rest, as well as other neurological problems resulting from damage to the substantia nigra as well as the cerebral cortex (15). Human parkinsonism has been further linked to immune activation through neurotrophic viral infection and, in particular, infection by the human influenza virus. Individual cases of viral infection leading to neuropathology and death have been reported, as well as increased incidence of Parkinson's disease following pandemic flu, such as experienced in 1918 (16).

At present, little is known about how *Lrrk2* modifies the gene expression program induced by innate signaling pathways and how this might contribute to Parkinson's disease initiation and progression. In this study, we used high-resolution transcriptomics to compare gene expression in primary macrophages derived from WT and *Lrrk2*-deficient mice. This analysis reveals that LRRK2 modulates the expression of a small subset of genes that are involved in chemotaxis and membrane remodeling.

## Results

### mRNA sequencing, quality control, and read mapping

We used RNA-Seq to determine how LRRK2 modifies gene expression in primary bone marrow–derived macrophages from WT and *Lrrk2*^−/−^ mice. If valid comparisons are to be made, then it is necessary to confirm the similarity in the nature and purity of macrophage cultures before RNA extraction and RNA-Seq. One day prior to RNA extraction, a portion of differentiated macrophages was prepared for flow cytometry analysis and stained for various cell surface markers: CD11b for cells of the myeloid lineage, F4/80 for mouse macrophages, and CD11c for monocyte-derived cells, including macrophages (17). These markers revealed no significant differences in the differentiation state of the cells, with uniform expression of CD11b and highly similar expression levels of F4/80 and CD11c. CD11c surface expression in *Lrrk2* KO macrophages displayed a slightly higher level of variability between cultures than equivalent WT cells (Fig. S1). Overall, cultures were considered similar enough to proceed with differential gene expression analysis.

We then treated WT and *Lrrk2*^−/−^ macrophages with either LPS or muramyl dipeptide (MDP), activators of the TLR4- and NOD2-mediated innate responses, respectively. After 2 h of stimulation, RNA was extracted for mRNA sequencing. A mean read depth of over 22.2 × 10^6^ reads/sample was achieved with a range of 16.0 × 10^6^ to 24.9 × 10^6^ reads/sample (Table S1). Reads were of high quality, requiring a mean of less than 0.1% of reads to be trimmed during quality control. A mean of 87.5% of reads could be unambiguously mapped to gene-encoding regions of the genome. Therefore, by comparing the frequency of reads per gene between samples, relative levels of expression could be determined. These datasets were then analyzed for differential gene expression.

### Differential gene expression

**Figure 1.**
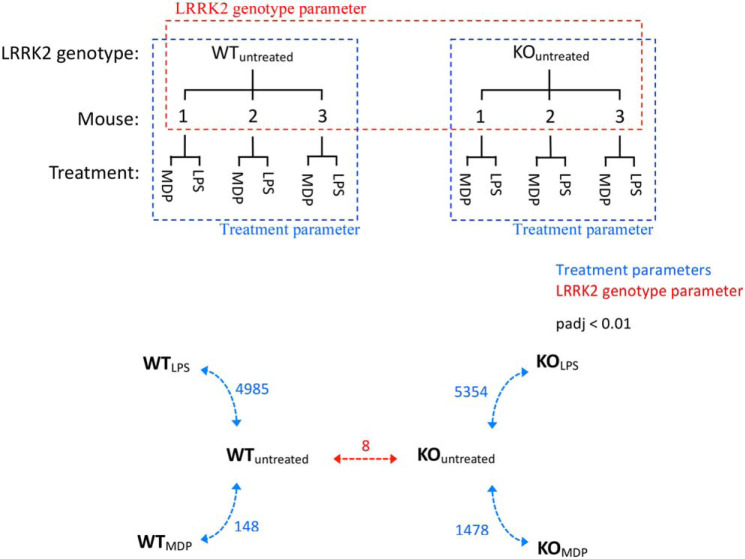
**Differential gene expression analysis.** *a*, *Lrrk2* genotype (*red*) and treatment with innate immune stimuli (*blue*) are considered separately in these experiments. *b*, quantification of differentially expressed genes. *KO* refers to the *Lrrk2* KO genotype. *Numbers* refer to differentially expressed genes between annotated samples (padj < 0.01).

Datasets of mapped counts were interrogated for differences in each *Lrrk2* genotype upon innate immune stimulation as well as for underlying differences between genotypes in unstimulated cells (Fig. 1*a*). DEseq2 (18) determines a statistical model accounting for variance in counts per gene and base mean of counts, allowing the statistical significance of apparent differences in gene expression to be estimated. This analysis revealed that LPS treatment caused differential expression of 4985 and 5354 genes in WT and *Lrrk2*^−/−^ macrophages, respectively. In contrast, MDP treatment resulted in 1483 significantly differentially expressed genes in WT macrophages compared with 1478 genes in *Lrrk2* KO macrophages (Fig. 1*b*).

In the absence of stimulation, only eight genes were significantly differentially expressed (Table 1). One of these is *Lrrk2*, a result confirmed by qPCR. The lesion in the *Lrrk2* KO mouse deletes part of exon 1 and exon 2, leading to termination at an out-of-frame stop codon in exon 3. As the *Lrrk2* transcription unit has 51 exons, this should lead to nonsense-mediated decay of the transcript. The level of transcript measured is about 50% of the WT, which indicates that nonsense-mediated decay is inefficient in this case (19). Other genes regulated include *kif21a*, a member of the kinesin family of motor proteins; *camk2b*, a calcium/calmodulin-responsive protein kinase; *cd59a*, a regulator of the membrane attack complex in mice; and *nnt*, a NAD(P) transhydrogenase with implications in defense against oxidative stress. Very little is known about the *lrmda* gene except that it consists of a region of LRRs. The remaining results are not represented at the protein level and so are unlikely to have relevance to the current study. The gene detected as being of the highest significance, *gm14150*, is described as a pseudogene, produced by incorporation of reverse-transcribed mRNA into the genome, whereas *gm44305* is a retained intron. These are likely not differentially expressed genes but pre-existing genomic differences between strains (20).

**Table 1:** **Differentially expressed genes between unstimulated macrophages** Shown are genes with padj < 0.01 in *Lrrk2* KO/WT pBMDM cells. Genes not represented at the protein level are displayed in boldface.

Ensembl Gene ID	BaseMean	-Fold Change (KO/WT)	Padj	Gene Symbol
**ENSMUSG00000082809**	**177.98**	**5.65**	**2.52E − 92**	**Pseudogene Gm14150**
ENSMUSG00000063458	83.02	0.44	3.99E − 20	Lrmda
ENSMUSG00000022629	31.47	1.93	1.07E − 14	Kif21a
**ENSMUSG00000105703**	**89.25**	**2.01**	**1.94E − 13**	**Gm43305**
ENSMUSG00000036273	137.37	0.54	8.10E − 10	Lrrk2
ENSMUSG00000057897	58.28	1.66	1.17E − 07	Camk2b
ENSMUSG00000032679	297.27	1.55	1.02E − 04	Cd59a
ENSMUSG00000025453	784.61	1.43	9.56E − 04	Nnt

**Figure 2.**
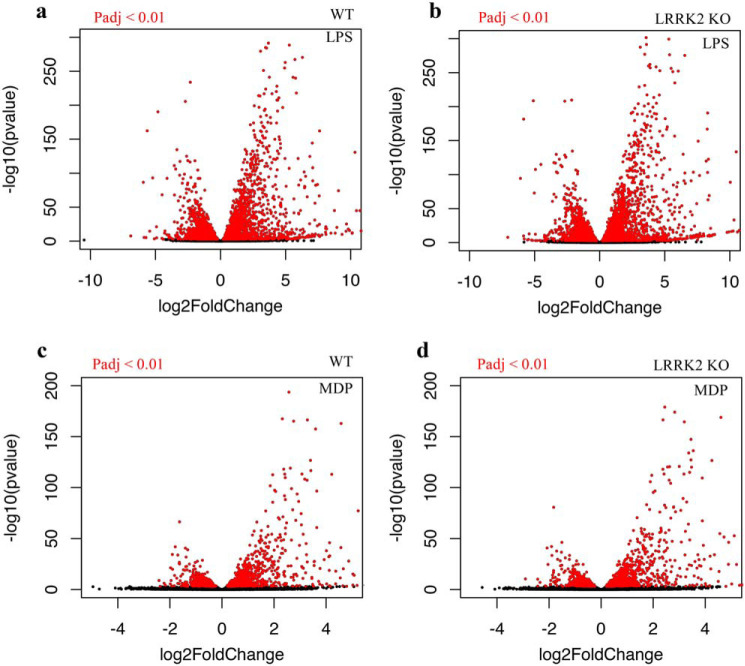
**Volcano plot visualization of transcriptional gene responses.** *a–d*, dots represent individual genes. *Red* indicates padj < 0.01. *a* and *c*, WT pBMDM cells. *b* and *d*, *Lrrk2* KO pBMDM cells.

The broad characteristics of the MDP and LPS responses as well as similarities and differences between WT and *Lrrk2*-deficient macrophages in their response to innate immune activation were visualized with volcano plots (Fig. 2). In LPS and MDP experiments, a greater number of genes were up-regulated than down-regulated. LPS treatment led to 3192 genes being up-regulated and 2696 down-regulated; MDP treatment caused 1020 genes to be up-regulated and 676 down-regulated (Fig. 1). Furthermore, quantification confirmed that a greater number of genes was differentially expressed upon LPS treatment in *Lrrk2*-deficient macrophages than WT macrophages. Perhaps the most interesting observation from this analysis is that a single gene was found to be down-regulated in WT macrophages and up-regulated in *Lrrk2*^−/−^ macrophages upon treatment with LPS. Transcription of this gene, *Rapgef3*, is almost halved upon LPS treatment in WT macrophages while being increased just over 7-fold in *Lrrk2*-null macrophages, a complete reversal in transcriptional regulation upon loss of LRRK2.

### Two parameter analysis identifies a small subset of differentially responding genes that are involved in cell motility and chemotaxis

**Figure 3.**
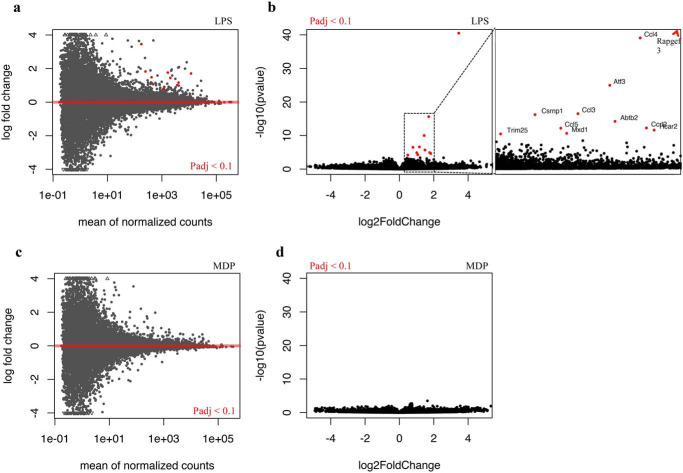
**Visualization of differentially responding genes.** *a–d*, -fold changes are ligand-treated gene expression levels (*Lrrk2* KO/WT). *Dots* represent individual genes. *Red dots* indicate padj < 0.1.

In contrast to conventional differential gene expression analysis, two-parameter analysis provides an alternative method to identify differentially responding genes between genotypes (21) (RRID: SCR_012802). This method revealed 11 genes with significantly different responses to LPS as between WT and *Lrrk2*^−/−^ macrophages (adjusted P-value (Padj) < 0.1) (Fig. 3 and Table 2). All differentially responding genes showed an increased level of transcription upon LPS stimulation in *Lrrk2* KO cells compared with WT cells. *Rapgef3* with a Padj value of 5 × 10^−37^ and a -fold change of about 11 was the gene most strongly regulated by the presence of Lrrk2. Four other genes identified encode either chemokine ligands (*Ccl3*, *Ccl4*, and *Ccl5*) or receptors (*Ccrl2*) that mediate chemotactic responses, suggesting a common theme of regulated motility (see “Discussion”).

**Table 2:** **Differentially responding genes in LPS stimulated macrophages** Shown are genes with padj < 0.1 in LPS-treated LRRK2 KO/WT pBMDM cells.

Ensembl Gene ID	BaseMean	-Fold Change (KO/WT)	Padj	Gene Symbol
ENSMUSG00000022469	176.34	10.97	4.98E − 37	Rapgef3
ENSMUSG00000018930	11796.25	3.26	1.94E − 12	Ccl4
ENSMUSG00000026628	2039.03	2.72	5.58E − 07	Atf3
ENSMUSG00000000982	4095.93	2.24	1.02E − 03	Ccl3
ENSMUSG00000032515	1117.61	1.73	1.07E − 03	Csrnp1
ENSMUSG00000032724	417.68	2.80	5.72E − 03	Abtb2
ENSMUSG00000035042	3664.77	2.02	2.75E − 02	Ccl5
ENSMUSG00000043953	1652.79	3.39	2.75E − 02	Ccrl2
ENSMUSG00000045502	249.85	3.55	4.19E − 02	Hcar2
ENSMUSG00000001156	477.83	2.09	9.07E − 02	Mxd1
ENSMUSG00000000275	8075.68	1.40	9.83E − 02	Trim25

**Figure 4.**
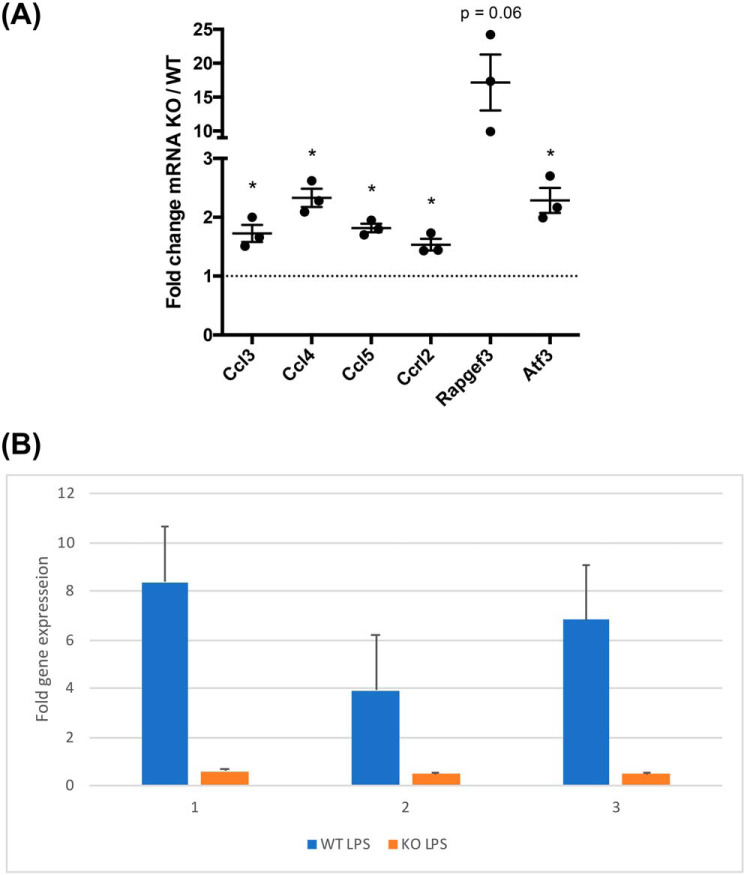
**Transcript levels upon LPS stimulation.** *a*, qRT-PCR with β-actin and GAPDH as housekeeping genes. Each point is a different *Lrrk2* KO pBMDM sample compared with three different WT pBMDM samples. *Error bars* are S.E. Paired two-tailed t tests compared with a value of 1 (* *p* < 0.05). *b*, equal aliquots of WT (*blue columns*) and *Lrrk2*^−/−^ (*orange columns*) macrophages were incubated for 4 h with or without the GSK2578215A inhibitor (1.3 μm) and then stimulated with LPS for 2 h. *Rapgef3* RNA was then quantified by qRT-PCR. WT untreated cells have active Lrrk2, and therefore *Rapgef3* expression is relatively inhibited compared with the equivalent treated cells, whereas mutant macrophages have similar levels of *Rapgef3* transcript in treated and control samples. Results are presented for three biological replicates.

To confirm the results of the RNA-Seq experiments, qRT-PCR was used to directly measure the level of six of the 11 genes identified in WT and *Lrrk2*^−/−^ macrophages (Fig. 4*A*). This confirms the results of the RNA-Seq analysis for *Rapgef3*, chemokines, and the transcription factor *atf3*. We next asked whether chemical inhibition of the LRRK2 kinase also induces expression of *Rapgef3.* We treated WT and *Lrrk2*^−/−^ macrophages with the highly specific inhibitor GSK2578215A (22). *Rapgef3* was induced about 6-fold in treated WT macrophages compared with untreated controls. In contrast, no differences in expression level were detected when *Lrrk2* mutant macrophages were treated with GSK2578215A relative to the untreated controls (Fig. 4*B*). Thus, Lrrk2 kinase activity is required for the observed regulation of *Rapgef3* gene expression, consistent with the results of the RNA-Seq analysis.

In contrast to LPS, MDP treatment identified no significant differentially responding genes (Fig. 3, *c* and *d*). This aligns with the less immunogenic nature of MDP compared with LPS stimulation and demonstrates the high stringency of the two-parameter method.

### Elevated levels of EPAC-1 protein in activated macrophages lacking LRRK2

**Figure 5.**
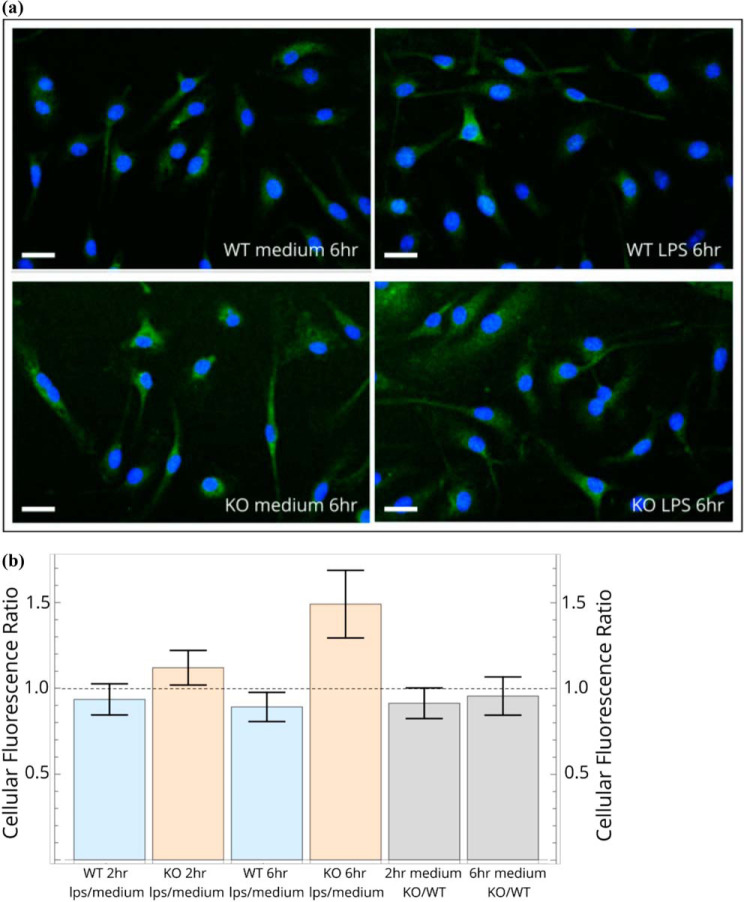
**Elevated levels of EPAC-1 in activated macrophages lacking LRRK2.** *a*, immunofluorescence images of cells stained with Alexa 488–conjugated anti-EPAC1 mAb (*green*) and DAPI (*blue*). *b*, projection image analysis of untreated and LPS-stimulated macrophages. The ratios of average fluorescence per cell in LPS treated *versus* medium were calculated at each time point for each genotype (see “Experimental procedures”). *Scale bars* = 20 μm.

To determine whether *Rapgef3* mRNA and Epac-1 protein levels are correlated, we stained Lrrk2-deficient and WT macrophages with a fluorescent mAb specific for Epac-1. As shown in Fig. 5*a*, Epac-1 is ubiquitous and, in many cells, distributed in the expected punctate, perinuclear pattern (Fig. S2). We then quantified protein levels. 6 h after treatment with LPS, Lrrk2-deficient cells had significantly higher levels of Epac-1, consistent with the RNA-Seq and qPCR results (Fig. 5*b*). We were unable to further validate these observations using Western blotting, as the available antibodies are insufficiently specific or sensitive in this assay.

### A sustained inflammatory response in LRRK2 knock out mice

**Figure 6.**
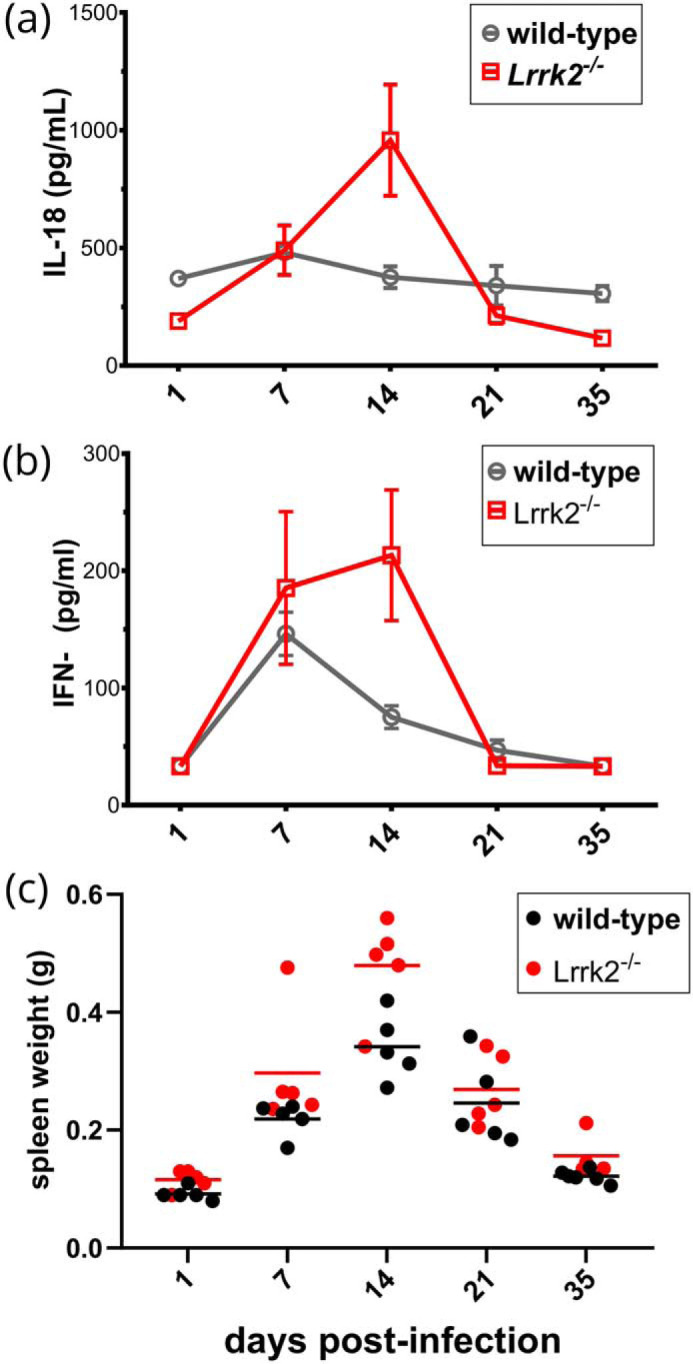
**LRRK2 dampens inflammation in a subclinical infection model.** *a–c*, WT and LRRK2^−/−^ (*n* = 5) mice were infected with *S. enterica* serovar Typhimurium 525P. IL-18 (*a*), IFN-γ (*b*), and spleen weight (*c*) were measured. Significance was evaluated with a Mann–Whitney test. Differences in IL-18 were significant (day1: *, *p* = 0.0159; day 14: *, *p* = 0.0159; day 35, **, *p* = 0.0095). IFN-γ, although elevated on day 14, was not significant at the 95% confidence level (*p* = 0.09). Spleen weight was significant on day 14 (*, *p* = 0.0317).

To explore whether LRRK2 affects innate immune function, we used a model of subclinical bacterial infection. WT and *Lrrk2*-deficient mice were infected with *Salmonella enterica* serovar Typhimurium, and three markers of inflammation were measured: IL-18, IFN-γ, and splenomegaly. As shown in Fig. 6 *Lrrk2*^−/−^ mice had elevated levels of IL-18 and IFN-γ compared with controls 14 days after challenge. These results are statistically significant (*p* < 0.05, with the exception IFN-γ at 14 days, *p* = 0.06; see legend for Fig. 6). This indicates that absence of Lrrk2 causes an enhanced inflammatory response.

## Discussion

In this study, we investigated how Lrrk2 modifies inflammatory signaling mediated by LPS and MDP. We identified a small subset of genes that are activated by LPS in macrophages that lack Lrrk2 but not in WT control cells. About half of these molecules are involved in cell migration, motility, and chemotaxis. Of particular note is the guanine nucleotide exchange factor (GEF) *Rapgef3*, by far the most strongly induced gene identified, with an 11- to 15-fold increase in transcription in the absence of Lrrk2. *Rapgef3* is also the only gene that is down-regulated in WT macrophages but up-regulated in *Lrrk2*^−/−^ cells as compared with unstimulated control cells. *Rapgef 3* is also derepressed by treatment of macrophages with the highly specific kinase inhibitor GSK2578215A (22), indicating that inter- or intramolecular phosphorylation is required.

*Rapgef3* encodes Epac-1, a GEF that mediates cAMP-dependent activation of the small G-protein Rap1. Epac-1 promotes Rap-1 GDP–GTP exchange, leading to cell adhesion, cell polarization, and enhanced leukocyte chemotaxis (23, 24). On the other hand, another study found that LPS treatment paralyzes monocyte chemotaxis, an effect that requires activated Rap-1 (25). It is thus likely that LRRK2 indirectly regulates the mobility of macrophages and microglia that have been activated by innate stimuli such as LPS. An attractive hypothesis is that dominant PD-associated LRRK2 alleles, such as G2019S, that have higher constitutive kinase activity may regulate the motility of macrophages by further suppressing EPAC-1 levels. In that regard, a recent study found that G2019S microglia and LRRK2^−/−^ cells, when activated by ADP, have retarded and enhanced motility, respectively, compared with WT control cells. G2019S microglia also have an impaired ability to isolate brain injury (26). These authors present evidence of involvement of focal adhesion kinases; however, this may be indirect, and a possible role of the LRRK2/EPAC-1/RAP-1 axis should be investigated.

LRRK2 is part of an ancient and highly conserved pathway of directional motility (2). In the slime mold *Dictyostelium*, *gbpC* is one of 11 paralogs of LRRK2. *GbpC*-null cells are severely defective in chemotaxis because they cannot polarize cells effectively and have altered patterns of myosin phosphorylation that are probably mediated by activation of Rap1. It is interesting to also note that, in *Dictyostelium*, many LRRK2 paralogues encode GEFs within their modular structure.

As well as Epac1, four other messages that encode proteins with functions in chemotaxis are differentially expressed. Three are chemokines, and one is a chemokine receptor–like protein. CCL3 is also known as macrophage inflammatory protein 1, and CCL4 is also known as MIP-1α and MIP-1β. CCL5 is also known as regulated on activation, normal T cell expressed and secreted (RANTES). These chemokines are all members of the CC chemokine/receptor family and share a common receptor in CCR5. CCL3 and CCL5 may also bind CCR1, whereas CCL5 binds a further receptor, CCR3 (27). These chemokines are all classified as pro-inflammatory, meaning they are induced by inflammatory stimuli to recruit inflammatory cells to a site of inflammation, as opposed to homeostatic chemokines, which are constitutively expressed in certain tissues (28). CCRL2 is chemokine receptor–like protein, with over 40% sequence identity to CCR1, CCR2, CCR3, and CCR5 (29) and highest amino acid sequence similarity to CCR1. Interestingly, CCRL2 has been reported as a noncanonical receptor for CCL5 (30) as well as CCL19 and chemerin (31). A microarray screen of unstimulated mouse microglia has shown that CX3CR1, a noncanonical chemokine receptor of fractalkine, expressed exclusively in microglia, is up-regulated by knockout of LRRK2 (32). This reaffirms that chemokine responses may be involved in LRRK2 biology in diverse immunological contexts.

Message encoding the transcription factor ATF3 is induced by 3-fold in the absence of LRRK2 and is a negative regulator of pro-inflammatory TLR4 signaling, a part of the LPS induced negative feedback loop (33, 34). Thus, it is possible that G2019S mutation of LRRK2 might down-regulate ATF3, resulting in attenuated negative feedback of TLR4 signaling, enhanced inflammation, and greater neuronal stress. Other transcription factor messages identified encode MXD1 and CSRNP1. MXD1 acts in a network with MYC and MAX, forming the MYC/MAX/MXD1 axis (33). MXD1 is in competition with MYC for binding of MAX, leading to a mixture of MXD1/MAX and MYC/MAX dimers controlling transcriptional output. In the context of cancer, MYC signaling affects cell adhesion and cell shape and reduces cell migration through modulation of the actin cytoskeleton (35). Finally, CSRNP1 is a transcription factor that is up-regulated by Axin as well as inflammatory stimuli in the form of IL-2 (36, 37). Axin is a negative regulator of the Wnt signaling pathway, acting to sequester the transcription factor β-catenin to the cytoplasm (38).

Two transcripts identified have been linked directly to Parkinson's disease. Abtb2 encodes ankyrin-rich BTB/POZ domain containing protein-2 (BPOZ-2). This protein causes inhibition of α-synuclein aggregation (39). Lentiviral delivery of the BPOZ-2 gene appears to stimulate autophagic clearance of α-synuclein, resulting in reduced α-synuclein pathology in basal ganglia. Another protein identified is the G-protein–coupled receptor HCAR2, otherwise known as niacin receptor 1. Niacin has been proposed as a treatment for Parkinson's disease, although evidence of efficacy is lacking (40, 41). Activation of HCAR2 in macrophages has an anti-inflammatory effect. Activation by niacin results in inhibition of CCL2-induced macrophage migration (42) as well as an inhibited response to LPS stimulation (43) or inflammatory cytokine release (44).

Previous studies of LRRK2 using kinase inhibitors have revealed immunological functions. However, these results should be treated with caution because of significant off-target effects that act on the extracellular signal-regulated kinase pathway (45). We therefore used a subclinical infection model to test for inflammatory phenotypes in *Lrrk2*^−/−^ mice. Mice were infected with *S. enterica* serovar Typhimurium, and this revealed that Lrrk2 significantly dampens the inflammatory response. In this model, IL-18 comes most likely from myeloid cells, particularly monocytes, and the principal inflammatory activator of *Salmonella* is LPS. This suggests that the observed differences in gene expression reported here cause subtle but significant changes in the inflammatory response *in vivo* that may be caused by alterations in the chemotactic capacity of monocytes and macrophages.

It is interesting that only a small number of differentially expressed genes were observed between *Lrrk2* genotypes under resting conditions. This shows that Lrrk2 only exerts an effect on the macrophage transcriptome under stimulated conditions. This finding also reflects work by another group that identified no changes in gene expression in unstimulated human fibroblasts or brain tissue between G2019S LRRK2 carriers and controls (46).

In conclusion, this study identifies the control of directional motility and chemotaxis of macrophages by LRRK2 as a potentially critical mechanism in the etiology of PD. It suggests that the function and regulation of the LRRK2/EPAC-1/RAP1 axis and how this impacts pro- and anti-inflammatory properties of microglia should be investigated. If active EPAC-1/RAP1 confers a neuroprotective phenotype on microglia, then EPAC-1–specific agonists, such as the cAMP analog 8-(4-chloro-phenylthio)-2′-*O*-methyladenosine-3′,5′-cyclic monophosphate, may have therapeutic value in PD (47).

## Experimental procedures

### Mice, genotyping, and cell culture

WT C57BL/6J mice were obtained from Charles River. LRRK2^−/−^ B6.129X1(FVB)-Lrrk2^tm1.1Cai^/J mice were obtained from The Jackson Laboratory (48). All mouse strains were bred independently. All work involving live animals complied with University of Cambridge Ethics Committee regulations and was performed under Home Office Project License 80/2572. DNA from ear snips of *Lrrk2*^−/−^ B6.129X1(FVB)-Lrrk2^tm1.1Cai^/J mice was isolated for genotyping using the Phire Animal Tissue Digest PCR Kit (Thermo Fisher Scientific). Genotyping PCR was carried out in accordance with recommendations from The Jackson Laboratory. Genotyping PCR products were run on a 1% agarose gel.

For differentiation and culture of primary bone marrow–derived macrophages (pBMDMs), mice were killed between 8 and 16 weeks of age by cervical dislocation, the skin was sterilized with 70% ethanol, and the legs were removed. Under sterile conditions, the tibiae and femora were isolated and cleaned of muscle, and the proximal and distal epiphyses were cut away. Bone marrow was flushed out of the bone using primary growth medium (DMEM, Thermo Fisher Scientific) supplemented with 10% FCS (Thermo Fisher Scientific), 20% L929 conditioned medium, and 8 mm l-glutamine (Sigma-Aldrich). Isolated cells were centrifuged at 300 × *g* for 10 min at 15 °C, resuspended in 60 ml of growth medium, and allowed to grow at 37 °C in 5% CO_2_. Cells were supplemented with a further 60 ml of growth medium after 2 days, and the medium was replaced every 3 days. All experiments were performed on cells between 6 and 11 days after initial bone marrow isolation. Live cell counts were performed using a hemocytometer with trypan blue staining (Sigma-Aldrich).

### Animal infection and data collection

*S. enterica* serovar Typhimurium strain M525P (49), a strain of intermediate virulence, was used to establish subclinical infection *in vivo*. In particular, overnight (stationary phase) bacterial cultures were first washed and resuspended in Dulbecco's PBS (Sigma) and then diluted to the desired dose. WT and *Lrrk2*^−/−^ mice were subsequently challenged intravenously with 0.2 ml of the bacterial suspension. The exact dose, as determined by serial dilution and plating the inoculum on LB plates before and after infection, was 1.3 × 10^4^ cfu/mouse. Mice were bled and then euthanized at certain intervals after the initial challenge. Their spleens were aseptically removed and weighed. Mouse serum was analyzed via ELISA for levels of IL-18 (MBL International) and IFN-γ (DuoSet Development Kit, R&D Systems).

### Flow cytometry

1 × 10^6^ cells/well were plated in 12-well tissue culture plates and left to adhere overnight at 37 °C in 5% CO_2_. Cells were then resuspended into MACS buffer (PBS supplemented with 2% FCS and 1 mm EDTA (Merck)) and spun at 300 × *g* for 6 min in a conical-bottom 96-well plate (Thermo Fisher Scientific). To block Fc-mediated reactions, cells were resuspended in MACS buffer supplemented with 1:100 rat anti-mouse CD16/CD32 monoclonal antibody (93 clone, eBioscience) and incubated at 4 °C for 15 min. Cells were spun at 300 × *g* for 6 min and resuspended in MACS buffer supplemented with rat anti-F4/80 conjugated with FITC, hamster anti-CD11c conjugated with phycoerythrin, and rat anti-CD11b conjugated with PerCP-cyanine5.5. Staining was performed for 30 min at 4 °C. Cells were then centrifuged at 300 × *g* for 6 min, resuspended in MACS buffer three times to remove unbound antibody, spun at 300 × *g* for 6 min, and resuspended in MACS buffer supplemented with 2% methanol-free formaldehyde (Thermo Fisher Scientific) to fix. Fixed cells were analyzed using an Attune NxT acoustic focusing cytometer (Life Technologies) for triple-labeling experiments.

### RNA-Seq and transcriptomics data analysis

Bone marrow was isolated from 16-week-old female mice housed in the same facility for this study. 3 × 10^6^ cells/well were plated in Greiner 6-well tissue culture plates (Sigma-Aldrich) a day prior to RNA extraction and left to adhere overnight at 37 °C in 5% CO_2_. Where appropriate, cells were then treated with 100 ng/ml ultrapure LPS from *Escherichia coli* O111:B4 or 10 μg/ml MDP. LPS was sonicated prior to application to cells. After 2-h incubation at 37 °C in 5% CO_2_, cells were washed in PBS and scraped into PBS at 4 °C. RNA was isolated using the RNeasy Mini Kit in combination with QIAshredder cell homogenization, following the manufacturer's instructions. To remove genomic DNA, extracted RNA was DNase-treated using the TURBO DNA-Free Kit. Resulting RNA was analyzed using a Nanodrop 1000 spectrophotometer. Samples with *A*_260/230_ < 1.8 were further purified with the RNeasy MinElute Cleanup Kit (Qiagen). Samples were then flash-frozen in liquid nitrogen, and RNA was quantified using a Qubit fluorometer (Thermo Fisher Scientific). RNA integrity was verified using a 2100 Bioanlyser (Agilent Genomics), and mRNA library preparation was performed using the TruSeq Stranded mRNA Library Prep Kit (Illumina) with quality control by a 2200 Tapestation (Agilent Genomics). High-output sequencing runs of single-end 75-bp read length were performed on NextSeq500 (Illumina) using the NextSeq 500/550 High Output v2 Kit (75 cycles, Illumina). A minimum read depth of 18 × 10^6^ reads/sample was achieved. Read preprocessing and mapping with quality control were performed using a standard pipeline. The Ensembl Mus_musculus.GRCm38.dna.primary_assembly.fa (release 84) reference genome file was used to map reads using the annotated transcripts from Ensembl Mus_musculus.GRCm38.84.gtf. Differential gene expression analysis was performed using DESeq2. Analysis was performed as a paired comparison experiment for each treatment group, as comparisons between genotype were made between different samples of different mice (unpaired), whereas comparisons of treated *versus* untreated samples were made using samples from the same mice (paired). A target frame and design matrix were adapted from an analogous scenario laid out in the EdgeR user guide, section 3.5: “Comparisons both between and within subjects” (21). This analysis enabled simple differential gene expression analysis between genotypes and two-parameter analysis to compare responses of each genotype with innate immune stimuli by interrogation of a target frame. This targets frame identifies each sample as belonging to a mouse (mouse.n) and each of these mice as being treated with LPS or MDP or left untreated (medium).

### Quantitative RT-PCR

qRT-PCR was performed using SensiFAST SYBR No-ROX One-Step Kit (Bioline) following the manufacturer's instructions, and appropriate primers were selected based on data submitted to the primer bank database (50). qRT-PCR reactions were performed using a Rotor-Gene Q (Qiagen), and quantification of -fold-changes of transcript were calculated using cycle threshold values accounting for reaction efficiency (51). For inhibitor studies, cells were pretreated with 1.3 μm GSK2578215A for 4 h. Following this, they were treated with 100 ng/ml LPS for a further 2 h. Cells were then washed with 1× PBS and harvested. Total RNA was used for qRT-PCR using the Luna One Step qRT-PCR kit (New England Biolabs), following the manufacturer's protocol.

### Immunofluorescence microscopy

Cells were fixed with 4% paraformaldehyde, permeabilized with 0.1% Triton X-100 (20 min), blocked by washing with 0.05% Tween 20 in PBS, followed by blocking for 30 min at 37 °C in 0.05% Tween 20 and 0.5% BSA. Cells were immunostained overnight at 4 °C with Alexa 488–conjugated anti-EPAC1 mAb (Abcam, ab201506). The Anti-Epac1 antibody is raised against human EPAC but to an epitope that is conserved in the mouse homolog. After staining, the slides were washed with blocking buffer (PBS) and then mounted in curing mountant with DAPI (Diamond Antifade, Thermo Fisher, P36966).

Fluorescence was detected under a FV1200 confocal microscope ×60 oil immersion objective with integration as 512 × 512 pixels, each 0.413 × 0.413 μm, with detector gain set for minimal saturated pixel count (4096 in the 13-bit intensity Olympus format). Z-stacks of 5 × 1-μm depth were collected upward from the coverslip to collect the total cellular fluorescence. Projections summing each pixel in each z series were calculated in ImageJ, and the image was converted to Flexible Image Transport System (FITS) image format to preserve the Olympus raw pixel intensity count data. Fields of view from medium-treated and LPS-treated cells for each genotype were collected in batches of 10 images as close in time as possible. As a result, intensity differences within a genotype were more reliable than comparison between genotypes. This approach was chosen so that the differing response of the cell types to LPS could be detected more accurately.

Projection image analysis was performed with Mathematica 11. Cell bodies were masked using the MorphologicalBinarize function extended by a border of 5 pixels (2 μm) for edge inclusion. Cell number was obtained from the binarized DAPI channel using the MorphologicalComponent function to give a count of the nuclei. EPAC1 immunofluorescence from the cytoplasm and nucleus compartments were summed, typically giving an intensity of millions of Olympus fluorescence units. Data from ∼200–300 cells in 20 fields of view were analyzed for each sample, and reported mean intensity is given in million units per cell. Cells cut by image borders were included in the pixel counts even when not containing the nucleus (as, on average, a cell should be cut by a border into two halves, one with and one without a nucleus). The mean total intensity per cell was calculated as a measure of EPAC1 expression with a standard error based on number of fields of view included (rather than the number of cells). Ratios of average fluorescence per cell in LPS-treated cells *versus* medium-treated cells were calculated at each time point for each genotype. In addition, the ratio of WT to knockout cells was calculated.

## Author contributions

D. R. L., M. F. S., C. E. B., and N. J. G. conceptualization; D. R. L., A. U., M. F. S., and L. J. H. formal analysis; D. R. L., A. U., P. T., M. F. S., L. J. H., and N. J. G. investigation; D. R. L. and N. J. G. writing-original draft; M. F. S., L. J. H., C. E. B., and N. J. G. supervision; M. F. S. methodology; C. E. B. and N. J. G. funding acquisition; C. E. B. and N. J. G. project administration; N. J. G. writing-review and editing.

## Supplementary Material

Supporting Information

## Footnotes

3 The abbreviations used are:

LRR: leucine-rich repeat
PD: Parkinson's disease
TLR: Toll-like receptor
LPS: lipopolysaccharide
MDP: muramyl dipeptide
qRT-PCR: quantitative RT-PCR
qPCR: quantitative PCR
GEF: guanine nucleotide exchange factor
pBMDM: primary bone marrow–derived macrophage
DAPI: 4′,6-diamidino-2-phenylindole.

## References

[1] Mills R. D., Mulhern T. D., Liu F., Culvenor J. G., and Cheng H. C. (2014) Prediction of the repeat domain structures and impact of parkinsonism-associated variations on structure and function of all functional domains of leucine-rich repeat kinase 2 (LRRK2). Hum. Mutat. 35, 395–412 10.1002/humu.22515 24470158

[2] Marín I., van Egmond W. N., and van Haastert P. J. (2008) The Roco protein family: a functional perspective. FASEB J. 22, 3103–3110 10.1096/fj.08-111310 18523161

[3] Russo I., Berti G., Plotegher N., Bernardo G., Filograna R., Bubacco L., and Greggio E. (2015) Leucine-rich repeat kinase 2 positively regulates inflammation and down-regulates NF-κB p50 signaling in cultured microglia cells. J. Neuroinflammation 12, 230 10.1186/s12974-015-0449-7 26646749 PMC4673731

[4] Di Fonzo A., Rohé C. F., Ferreira J., Chien H. F., Vacca L., Stocchi F., Guedes L., Fabrizio E., Manfredi M., Vanacore N., Goldwurm S., Breedveld G., Sampaio C., Meco G., Barbosa E., et al (2005) A frequent LRRK2 gene mutation associated with autosomal dominant Parkinson's disease. Lancet 365, 412–415 10.1016/S0140-6736(05)17829-5 15680456

[5] Nichols W. C., Pankratz N., Hernandez D., Paisán-Ruíz C., Jain S., Halter C. A., Michaels V. E., Reed T., Rudolph A., Shults C. W., Singleton A., Foroud T., and Parkinson Study Group-PROGENI Investigators (2005) Genetic screening for a single common LRRK2 mutation in familial Parkinson's disease. Lancet 365, 410–412 10.1016/S0140-6736(05)17828-3 15680455

[6] Gilks W. P., Abou-Sleiman P. M., Gandhi S., Jain S., Singleton A., Lees A. J., Shaw K., Bhatia K. P., Bonifati V., Quinn N. P., Lynch J., Healy D. G., Holton J. L., Revesz T., and Wood N. W. (2005) A common LRRK2 mutation in idiopathic Parkinson's disease. Lancet 365, 415–416 10.1016/S0140-6736(05)17830-1 15680457

[7] Greggio E., Civiero L., Bisaglia M., and Bubacco L. (2012) Parkinson's disease and immune system: is the culprit LRRKing in the periphery? J. Neuroinflammation 9, 94 22594666 10.1186/1742-2094-9-94 PMC3391996

[8] Gardet A., Benita Y., Li C., Sands B. E., Ballester I., Stevens C., Korzenik J. R., Rioux J. D., Daly M. J., Xavier R. J., and Podolsky D. K. (2010) LRRK2 is involved in the IFN-γ response and host response to pathogens. J. Immunol. 185, 5577–5585 10.4049/jimmunol.1000548 20921534 PMC3156100

[9] Thévenet J., Pescini Gobert R., Hooft van Huijsduijnen R., Wiessner C., and Sagot Y. J. (2011) Regulation of LRRK2 expression points to a functional role in human monocyte maturation. PLoS ONE 6, e21519 10.1371/journal.pone.0021519 21738687 PMC3124520

[10] Dzamko N., Inesta-Vaquera F., Zhang J., Xie C., Cai H., Arthur S., Tan L., Choi H., Gray N., Cohen P., Pedrioli P., Clark K., and Alessi D. R. (2012) The IκB kinase family phosphorylates the Parkinson's disease kinase LRRK2 at Ser935 and Ser910 during Toll-like receptor signaling. PLoS ONE 7, e39132 10.1371/journal.pone.0039132 22723946 PMC3377608

[11] Karin M. (1999) How NF-κB is activated: the role of the IκB kinase (IKK) complex. Oncogene 18, 6867–6874 10.1038/sj.onc.1203219 10602462

[12] Castaño A., Herrera A. J., Cano J., and Machado A. (2002) The degenerative effect of a single intranigral injection of LPS on the dopaminergic system is prevented by dexamethasone, and not mimicked by rh-TNF-α, IL-1β and IFN-γ. J. Neurochem. 81, 150–157 10.1046/j.1471-4159.2002.00799.x 12067227

[13] Iravani M. M., Leung C. C., Sadeghian M., Haddon C. O., Rose S., and Jenner P. (2005) The acute and the long-term effects of nigral lipopolysaccharide administration on dopaminergic dysfunction and glial cell activation. Eur. J. Neurosci. 22, 317–330 10.1111/j.1460-9568.2005.04220.x 16045485

[14] Qin L., Wu X., Block M. L., Liu Y., Breese G. R., Hong J. S., Knapp D. J., and Crews F. T. (2007) Systemic LPS causes chronic neuroinflammation and progressive neurodegeneration. Glia 55, 453–462 10.1002/glia.20467 17203472 PMC2871685

[15] Niehaus I., and Lange J. H. (2003) Endotoxin: is it an environmental factor in the cause of Parkinson's disease? Occup. Environ. Med. 60, 378 10.1136/oem.60.5.378 12709528 PMC1740529

[16] Jang H., Boltz D. A., Webster R. G., and Smeyne R. J. (2009) Viral parkinsonism. Biochim. Biophys. Acta 1792, 714–721 10.1016/j.bbadis.2008.08.001 18760350 PMC4642437

[17] Murray P. J., and Wynn T. A. (2011) Protective and pathogenic functions of macrophage subsets. Nat. Rev. Immunol. 11, 723–737 10.1038/nri3073 21997792 PMC3422549

[18] Love M. I., Huber W., and Anders S. (2014) Moderated estimation of fold change and dispersion for RNA-seq data with DESeq2. Genome Biol. 15, 550 10.1186/s13059-014-0550-8 25516281 PMC4302049

[19] Linde L., Boelz S., Neu-Yilik G., Kulozik A. E., and Kerem B. (2007) The efficiency of nonsense-mediated mRNA decay is an inherent character and varies among different cells. Eur. J. Hum. Genet. 15, 1156–1162 10.1038/sj.ejhg.5201889 17625509

[20] Akagi K., Li J., Stephens R. M., Volfovsky N., and Symer D. E. (2008) Extensive variation between inbred mouse strains due to endogenous L1 retrotransposition. Genome Res. 18, 869–880 10.1101/gr.075770.107 18381897 PMC2413154

[21] Robinson M. D., McCarthy D. J., and Smyth G. K. (2010) edgeR: a Bioconductor package for differential expression analysis of digital gene expression data. Bioinformatics 26, 139–140 10.1093/bioinformatics/btp616 19910308 PMC2796818

[22] Reith A. D., Bamborough P., Jandu K., Andreotti D., Mensah L., Dossang P., Choi H. G., Deng X., Zhang J., Alessi D. R., and Gray N. S. (2012) GSK2578215A; a potent and highly selective 2-arylmethyloxy-5-substitutent-*N*-arylbenzamide LRRK2 kinase inhibitor. Bioorg. Med. Chem. Lett. 22, 5625–5629 10.1016/j.bmcl.2012.06.104 22863203 PMC4208292

[23] Robichaux W. G. 3rd, and Cheng X. (2018) Intracellular cAMP sensor EPAC: physiology, pathophysiology, and therapeutics development. Physiol. Rev. 98, 919–1053 10.1152/physrev.00025.2017 29537337 PMC6050347

[24] Lorenowicz M. J., van Gils J., de Boer M., Hordijk P. L., and Fernandez-Borja M. (2006) Epac1-Rap1 signaling regulates monocyte adhesion and chemotaxis. J. Leukoc. Biol. 80, 1542–1552 10.1189/jlb.0506357 16940330

[25] Yi L., Chandrasekaran P., and Venkatesan S. (2012) TLR signaling paralyzes monocyte chemotaxis through synergized effects of p38 MAPK and global Rap-1 activation. PLoS ONE 7, e30404 10.1371/journal.pone.0030404 22347375 PMC3276499

[26] Choi I., Kim B., Byun J. W., Baik S. H., Huh Y. H., Kim J. H., Mook-Jung I., Song W. K., Shin J. H., Seo H., Suh Y. H., Jou I., Park S. M., Kang H. C., and Joe E. H. (2015) LRRK2 G2019S mutation attenuates microglial motility by inhibiting focal adhesion kinase. Nat. Commun. 6, 8255 10.1038/ncomms9255 26365310 PMC4647842

[27] Zlotnik A., and Yoshie O. (2000) Chemokines: a new classification system and their role in immunity. Immunity 12, 121–127 10.1016/S1074-7613(00)80165-X 10714678

[28] Turner M. D., Nedjai B., Hurst T., and Pennington D. J. (2014) Cytokines and chemokines: at the crossroads of cell signalling and inflammatory disease. Biochim. Biophys. Acta 1843, 2563–2582 10.1016/j.bbamcr.2014.05.014 24892271

[29] Migeotte I., Franssen J. D., Goriely S., Willems F., and Parmentier M. (2002) Distribution and regulation of expression of the putative human chemokine receptor HCR in leukocyte populations. Eur. J. Immunol. 32, 494–501 10.1002/1521-4141(200202)32:2<494::AID-IMMU494>3.0.CO;2-Y 11828366

[30] Yoshimura T., and Oppenheim J. J. (2011) Chemokine-like receptor 1 (CMKLR1) and chemokine (C-C motif) receptor-like 2 (CCRL2): two multifunctional receptors with unusual properties. Exp. Cell Res. 317, 674–684 10.1016/j.yexcr.2010.10.023 21056554 PMC3049852

[31] Akram I. G., Georges R., Hielscher T., Adwan H., and Berger M. R. (2016) The chemokines CCR1 and CCRL2 have a role in colorectal cancer liver metastasis. Tumour Biol. 37, 2461–2471 10.1007/s13277-015-4089-4 26383527

[32] Ma B., Xu L., Pan X., Sun L., Ding J., Xie C., Koliatsos V. E., and Cai H. (2016) LRRK2 modulates microglial activity through regulation of chemokine (C-X3-C) receptor 1-mediated signalling pathways. Hum. Mol. Genet 25, 3515–3523 10.1093/hmg/ddw194 27378696 PMC5179946

[33] Cascón A., and Robledo M. (2012) MAX and MYC: a heritable breakup. Cancer Res. 72, 3119–3124 10.1158/0008-5472.CAN-11-3891 22706201

[34] Gilchrist M., Thorsson V., Li B., Rust A. G., Korb M., Roach J. C., Kennedy K., Hai T., Bolouri H., and Aderem A. (2006) Systems biology approaches identify ATF3 as a negative regulator of Toll-like receptor 4. Nature 441, 173–178 10.1038/nature04768 16688168

[35] Liu H., Radisky D. C., Yang D., Xu R., Radisky E. S., Bissell M. J., and Bishop J. M. (2012) MYC suppresses cancer metastasis by direct transcriptional silencing of αv and β3 integrin subunits. Nat. Cell Biol. 14, 567–574 10.1038/ncb2491 22581054 PMC3366024

[36] Ishiguro H., Tsunoda T., Tanaka T., Fujii Y., Nakamura Y., and Furukawa Y. (2001) Identification of AXUD1, a novel human gene induced by AXIN1 and its reduced expression in human carcinomas of the lung, liver, colon and kidney. Oncogene 20, 5062–5066 10.1038/sj.onc.1204603 11526492

[37] Gingras S., Pelletier S., Boyd K., and Ihle J. N. (2007) Characterization of a family of novel cysteine-serine-rich nuclear proteins (CSRNP). PLoS ONE 2, e808 10.1371/journal.pone.0000808 17726538 PMC1950078

[38] Nakamura T., Hamada F., Ishidate T., Anai K., Kawahara K., Toyoshima K., and Akiyama T. (1998) Axin, an inhibitor of the Wnt signalling pathway, interacts with β-catenin, GSK-3β and APC and reduces the β-catenin level. Genes Cells 3, 395–403 10.1046/j.1365-2443.1998.00198.x 9734785

[39] Roy A., Rangasamy S. B., Kundu M., and Pahan K. (2016) BPOZ-2 gene delivery ameliorates α-synucleinopathy in A53T transgenic mouse model of Parkinson's disease. Sci. Rep. 6, 22067 10.1038/srep22067 26916519 PMC4768134

[40] Wakade C., and Chong R. (2014) A novel treatment target for Parkinson's disease. J. Neurol Sci. 347, 34–38 10.1016/j.jns.2014.10.024 25455298

[41] Wakade C., Chong R., Bradley E., Thomas B., and Morgan J. (2014) Upregulation of GPR109A in Parkinson's disease. PLoS ONE 9, e109818 10.1371/journal.pone.0109818 25329911 PMC4201464

[42] Lukasova M., Hanson J., Tunaru S., and Offermanns S. (2011) Nicotinic acid (niacin): new lipid-independent mechanisms of action and therapeutic potentials. Trends Pharmacol. Sci. 32, 700–707 10.1016/j.tips.2011.08.002 21944259

[43] Digby J. E., Martinez F., Jefferson A., Ruparelia N., Chai J., Wamil M., Greaves D. R., and Choudhury R. P. (2012) Anti-inflammatory effects of nicotinic acid in human monocytes are mediated by GPR109A-dependent mechanisms. Arterioscler. Thromb. Vasc. Biol. 32, 669–676 10.1161/ATVBAHA.111.241836 22267479 PMC3392598

[44] Zandi-Nejad K., Takakura A., Jurewicz M., Chandraker A. K., Offermanns S., Mount D., and Abdi R. (2013) The role of HCA2 (GPR109A) in regulating macrophage function. FASEB J. 27, 4366–4374 10.1096/fj.12-223933 23882124 PMC3804742

[45] Luerman G. C., Nguyen C., Samaroo H., Loos P., Xi H., Hurtado-Lorenzo A., Needle E., Stephen Noell G., Galatsis P., Dunlop J., Geoghegan K. F., and Hirst W. D. (2014) Phosphoproteomic evaluation of pharmacological inhibition of leucine-rich repeat kinase 2 reveals significant off-target effects of LRRK-2-IN-1. J. Neurochem. 128, 561–576 10.1111/jnc.12483 24117733

[46] Devine M. J., Kaganovich A., Ryten M., Mamais A., Trabzuni D., Manzoni C., McGoldrick P., Chan D., Dillman A., Zerle J., Horan S., Taanman J. W., Hardy J., Marti-Masso J. F., Healy D., et al (2011) Pathogenic LRRK2 mutations do not alter gene expression in cell model systems or human brain tissue. PLoS ONE 6, e22489 10.1371/journal.pone.0022489 21799870 PMC3142158

[47] Banerjee U., and Cheng X. (2015) Exchange protein directly activated by cAMP encoded by the mammalian rapgef3 gene: structure, function and therapeutics. Gene 570, 157–167 10.1016/j.gene.2015.06.063 26119090 PMC4556420

[48] Parisiadou L., Xie C., Cho H. J., Lin X., Gu X. L., Long C. X., Lobbestael E., Baekelandt V., Taymans J. M., Sun L., and Cai H. (2009) Phosphorylation of ezrin/radixin/moesin proteins by LRRK2 promotes the rearrangement of actin cytoskeleton in neuronal morphogenesis. J. Neurosci. 29, 13971–13980 10.1523/JNEUROSCI.3799-09.2009 19890007 PMC2807632

[49] Mastroeni P., Vazquez-Torres A., Fang F. C., Xu Y., Khan S., Hormaeche C. E., and Dougan G. (2000) Antimicrobial actions of the NADPH phagocyte oxidase and inducible nitric oxide synthase in experimental salmonellosis: II: effects on microbial proliferation and host survival *in vivo*. J. Exp. Med. 192, 237–248 10.1084/jem.192.2.237 10899910 PMC2193252

[50] Spandidos A., Wang X., Wang H., and Seed B. (2010) PrimerBank: a resource of human and mouse PCR primer pairs for gene expression detection and quantification. Nucleic Acids Res. 38, D792–D799 10.1093/nar/gkp1005 19906719 PMC2808898

[51] Pfaffl M. W. (2001) A new mathematical model for relative quantification in real-time RT-PCR. Nucleic Acids Res. 29, e45 10.1093/nar/29.9.e45 11328886 PMC55695

