# Supplementary material for: The Parkinson's disease–associated kinase LRRK2 regulates genes required for cell adhesion, polarization, and chemotaxis in activated murine macrophages
Source: J Biol Chem. 2020 Feb 28;295(31):10857–67. doi: 10.1074/jbc.RA119.011842 (PMC7397110; doi:10.1074/jbc.RA119.011842)
Supplement: Supporting Information [file supp_295_31_10857__index.html]

Parkinson’s disease–associated kinase LRRK2 regulates genes required for cell adhesion, polarization, and chemotaxis in activated murine macrophages — Role of LRRK2 in inflammation induced chemotaxis — The Parkinson's disease–associated kinase LRRK2 regulates genes required for cell adhesion, polarization, and chemotaxis in activated murine macrophages — The role of LRRK2 in inflammation-induced chemotaxis — Supporting Information 

# The Parkinson's disease–associated kinase LRRK2 regulates genes required for cell adhesion, polarization, and chemotaxis in activated murine macrophages

## Supporting Information

- Supporting Information (to be published online) - Fig S1, Fig. S2, Table S1
