# Supplementary material for: The Parkinson's disease–associated kinase LRRK2 regulates genes required for cell adhesion, polarization, and chemotaxis in activated murine macrophages
Source: J Biol Chem. 2020 Feb 28;295(31):10857–67. doi: 10.1074/jbc.RA119.011842 (PMC7397110; doi:10.1074/jbc.RA119.011842)
Supplement: Supporting Information [file supp_RA119.011842_157030_2_supp_482862_q6d376.pdf]

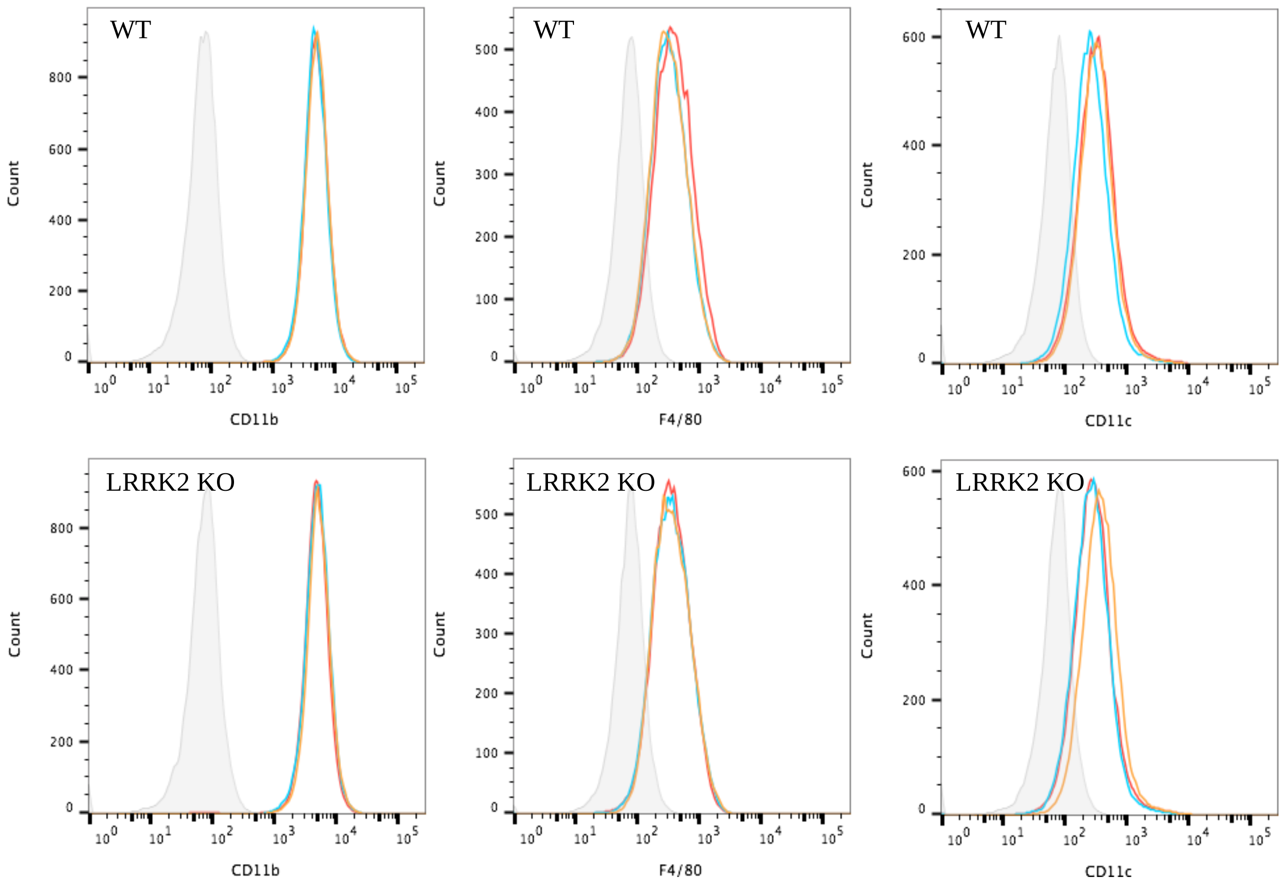

Marker:	Myeloid cells	Mouse macrophage	Monocyte derived cells

Figure S1: Analysis of macrophage cell markers. Flow cytometry analysis of macrophage cell surface markers. Each coloured, unfilled peak are stained cells of a macrophage population derived from a different mouse. Filled grey peaks are unstained cells derived from a single mouse.

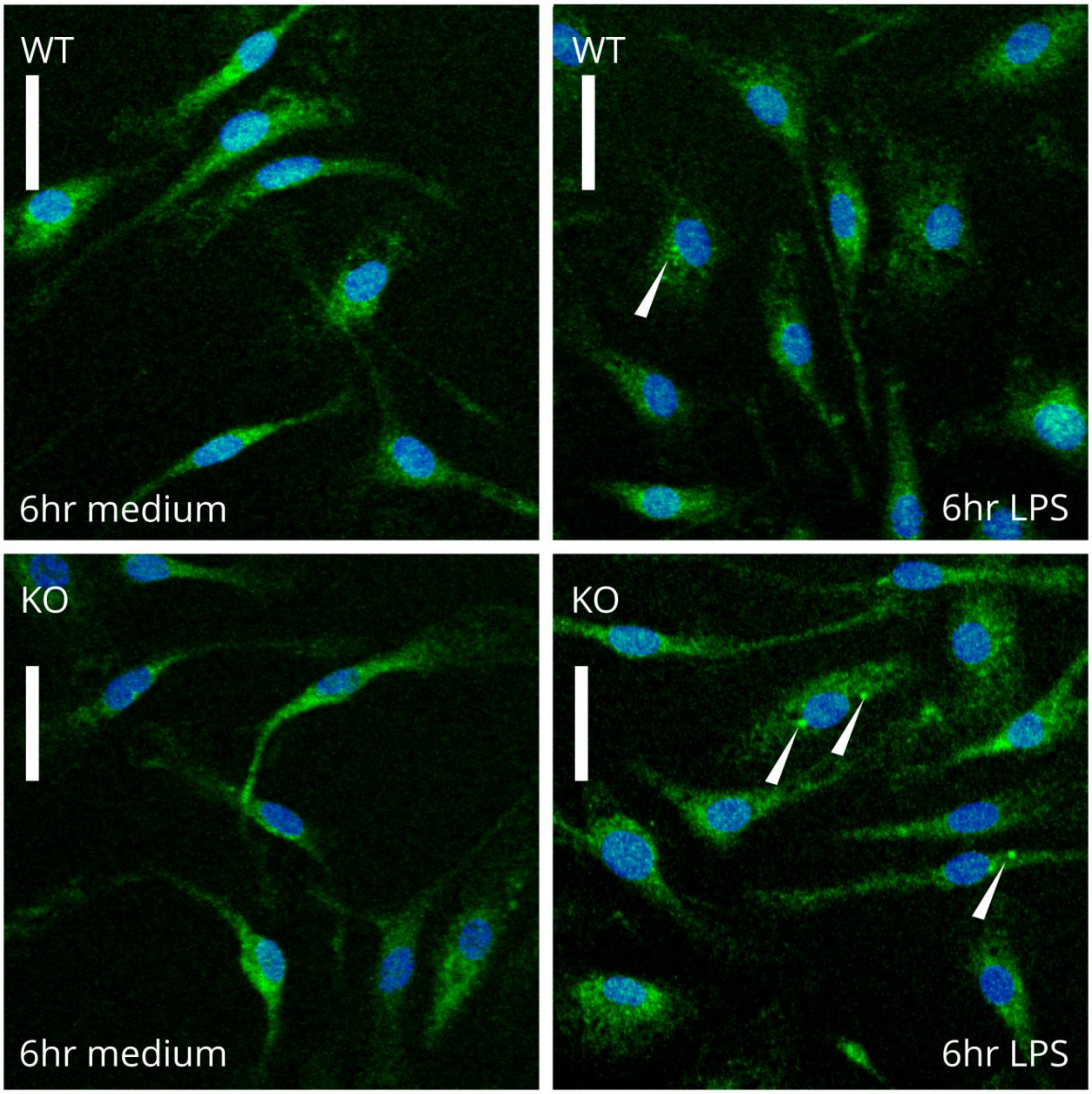

Supplementary Figure 2. Distribution of EPAC-1 in macrophages.
Higher magnification, enhanced contrast images of immunofluorescence-stained macrophage cells with Alexa-488 conjugated Anti-EPAC1 monoclonal antibody (green) and DAPI (blue). Macrophages are somewhat variable in form and show differing patterns of staining. However, some more elongated cells can show juxtanuclear endosomal punctae. Some marked punctate staining in the cytoplasm of LPS-activated cells was observed to correspond to endocytic vesicles visible in the dark-field images (not shown, but positions indicated by arrowheads). Scale bar corresponds to 20 µm.

			Read number		
Sample	LRRK2 Genotype	Treatment	Raw	Trimmed	Uniquely mapped to genes
1	KO	LPS	22,350,423	22,337,177	19,756,554
2	KO	MDP	20,864,607	20,852,101	18,035,498
3	KO	Media	24,584,270	24,574,608	21,373,819
4	KO	LPS	23,543,303	23,526,041	20,817,399
5	KO	MDP	23,633,755	23,624,109	20,888,105
6	KO	Media	20,997,179	20,979,364	18,387,880
7	KO	LPS	28,876,974	28,863,515	24,950,718
8	KO	MDP	21,917,338	21,889,839	18,953,616
9	KO	Media	22,083,849	22,073,008	19,232,352
10	WT	LPS	19,754,246	19,746,221	17,475,368
11	WT	MDP	23,022,129	23,009,814	19,887,489
12	WT	Media	23,088,368	23,069,013	19,908,328
13	WT	LPS	20,633,505	20,608,724	18,251,968
14	WT	MDP	21,367,108	21,352,531	18,693,589
15	WT	Media	25,560,513	25,549,470	22,693,200
16	WT	LPS	19,993,017	19,980,006	17,633,954
17	WT	MDP	18,222,162	18,215,079	16,008,576
18	WT	Media	20,019,319	20,006,085	17,641,459

Table S1: RNA sequencing quality control and mapping. Number of reads per sample after each step of quality control and read mapping is indicated.
